# Highly specific imaging of mRNA in single cells by target RNA-initiated rolling circle amplification†
†Electronic supplementary information (ESI) available: Additional experimental materials, methods, DNA sequences and supplementary figures and tables. See DOI: 10.1039/c7sc00292k



**DOI:** 10.1039/c7sc00292k

**Published:** 2017-03-07

**Authors:** Ruijie Deng, Kaixiang Zhang, Yupeng Sun, Xiaojun Ren, Jinghong Li

**Affiliations:** a Department of Chemistry , Key Laboratory of Bioorganic Phosphorus Chemistry & Chemical Biology , Beijing Key Laboratory for Microanalytical Methods and Instrumentation , Tsinghua University , Beijing 100084 , China . Email: jhli@mail.tsinghua.edu.cn

## Abstract

Detection of single-cell gene expression with high spatial and sequence resolution is a key challenge in single cell biology. Herein, we propose a robust method for the direct detection of mRNA, termed target RNA-initiated rolling circle amplification, which enables imaging of mRNA with single-nucleotide and near-single-molecule resolution in single cells. By utilizing a Splint R ligase capable of efficiently catalyzing the ligation of a padlock probe by the target RNA, the method can enable the efficient detection of mRNA without reverse transcription (detection efficiency over 20%). Meanwhile, attributed to the ligation-based recognition process, the method confers specificity sufficient to genotype mRNAs with one-nucleotide variations. The method has enabled the spatial mapping and correlation analysis of gene expression in single cells which could help us to elucidate the gene functions and regulatory mechanisms. This method offers an mRNA profiling ability with high spatial resolution and sequence specificity, thus is expected to be a single-cell analysis platform for not only investigating gene expression, but also potentially for analyzing single-nucleotide variants or mRNA alternative splicing at single-cell level.

## Abstract 2

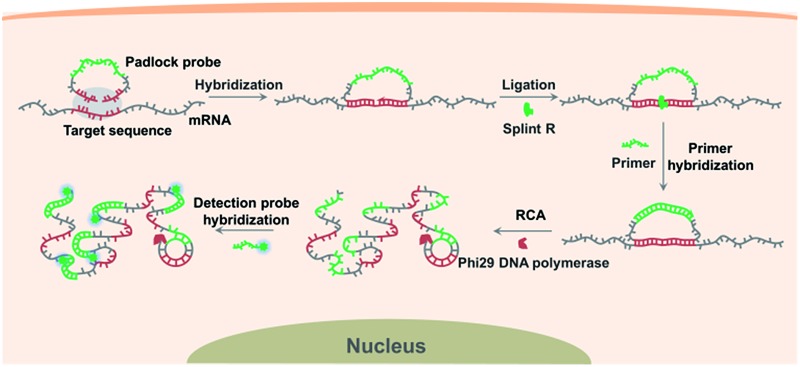
We report a robust method for the efficient imaging of mRNA with single-nucleotide and near-single-molecule resolution in single cells.

## Introduction

The gene expression program of a cell defines its function and identity. An abnormal gene expression may contribute to serious diseases including almost all kinds of cancer.^1,2^ As every cell expresses genes heterogeneously,^3–5^ the ability to map single-cell gene expression is essential for elucidating the genetic regulatory mechanism and the complexity and heterogeneity of disease development.

Advances in the imaging of single cells enable us to detect mRNA *in situ* with single-molecule resolution. Single molecule fluorescence *in situ* hybridization (smFISH) now is the most powerful tool for spatial mapping of mRNA in single cells.^6–8^ The ability of smFISH to precisely measure gene expression has allowed us to study the roles of gene regulation in a variety of biological processes, such as cell migration, development, and polarization.^9–13^ smFISH usually utilizes multiple fluorophore-labelled DNA probes simultaneously hybridized to the long target mRNA to obtain an enhanced fluorescence signal to distinguish individual mRNAs from the background.^6^ Thus, smFISH is constrained to target the long sequence and cannot resolve highly similar sequences.^14^ It is unable to be used for studying single nucleotide sequence variants and splice variation (in which it needs to target the conjugation region of exon–exon), and distinguishing gene family members with similar sequences.

As an alternative to smFISH, *in situ* rolling circle amplification (RCA) is a method that may potentially achieve highly specific imaging of mRNA in single cells with near-single-molecule resolution. RCA can achieve localized isothermal amplification to provide information about the localization of target molecules. It has enabled the *in situ* detection of widespread targets such as mitochondrial DNA,^15^ mRNA^14,16,17^ and miRNA.^18–20^ We have designed a seal probe and RCA method enabling the visualization of individual short miRNAs *in situ* in single cells.^18^ Chu *et al.* also developed a target-primed RCA method for the highly sensitive and selective *in situ* detection of miRNA expression.^19^ Nilsson and Lagunavicius’s groups pioneered the utilization of RCA coupling with a padlock probe for the imaging of individual RNAs and created protocols to detect and genotype mRNAs *in situ*.^14,16^ However, the RCA-based method still has several unresolved issues, which hinder its widespread application for mRNA detection. First, the efficiency of ligation of the padlock probes with the RNA template (the target mRNA) is low using the common ligase, T4 DNA ligase.^21^ Thus the direct detection of mRNA by RCA is with very low efficiency (1% or 4–7%, less than 7% of mRNAs in single cells can be detected and imaged).^16,22^ Reverse transcription is usually performed to convert target mRNA to cDNA,^14^ which will bring in variation in mRNA quantification^23,24^ and tremendously complicate the RNA detection process (Fig. S1 in the ESI†). Second, unlike miRNA with its small size (a length of ∼22 nucleotides, nt), mRNA has a long sequence (usually over 1000 nt) and complex secondary structures, it is difficult to be directly utilized as the primer to be amplified for RCA.^22^

Herein, we develop a robust method for the direct detection of mRNA, enabling highly efficiently imaging of mRNA with single-nucleotide and near-single-molecule resolution in single cells. We find Splint R, a recently discovered ligase, fits well for mRNA detection, and it was most recently used for the *in vitro* detection of miRNAs.^25,26^ The ligase can efficiently catalyze the ligation of the padlock probe by a RNA template,^21^ enabling the efficient detection of mRNA without reverse transcription (the detection efficiency is over 20%). In addition, instead of using mRNA as primer, we introduced an extra short DNA sequence to act as the primer to perform *in situ* RCA. Benefiting from the high specificity of the ligation-based method and *in situ* amplification, mRNA in single cells can be efficiently visualized with single-nucleotide and near-single-molecule resolution.

## Results and discussion

### Overview of the target RNA-initiated RCA method for the imaging of mRNAs in single cells

**Scheme 1:**
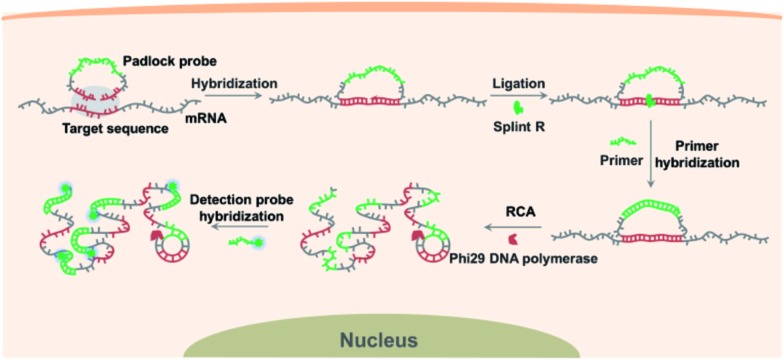
Schematic representation of the direct detection of mRNA in single cells by target RNA-initiated rolling circle amplification. A padlock probe was designed with the 5′- and 3′-terminal bases complementary to the target sequence of mRNA. Being hybridized with the target sequence, the target mRNA is specifically recognized by the padlock probe, initiating rolling circle amplification (RCA) to produce a long single stranded DNA RCA amplicon with hundreds of copies of the padlock probe. The RCA amplicon resulting from the individual mRNA recognition process can be visualized by being hybridized with the fluorophore-labelled detection probes.

Scheme 1 illustrates the procedures for the direct detection of mRNA in single cells by the target RNA-initiated RCA method. A padlock probe is designed with 5′- and 3′-terminal bases complementary to the target sequence of mRNA (partial mRNA sequence about 30 nt). Therefore, the padlock probe can be specifically ligated and circularized with the mRNA as the template in the presence of Splint R, a ligase which can efficiently catalyze the ligation of RNA-splinted DNA. The individual mRNA will initiate *in situ* RCA with the help of a short DNA primer, resulting in one-target-one-amplicon amplification. The RCA amplicon will become visible as a diffraction-limited fluorescent spot upon hybridization to the detection probes. Thus mRNA in single cells can be highly specifically recognized and visualized with near-single-molecule resolution.

### Detecting target RNA *in vitro* by target RNA-initiated RCA

**Fig. 1:**
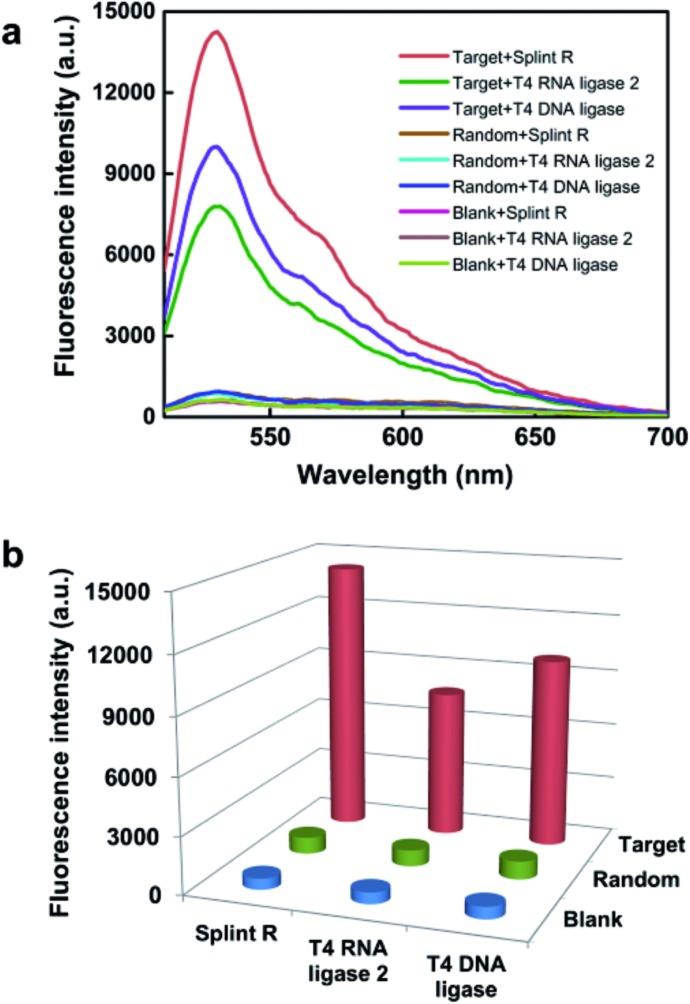
Detecting target RNA *in vitro* using target RNA-initiated RCA with different ligases. (a) Fluorescence emission spectra for the target RNA-initiated RCA reaction carried out using different ligases (Splint R, T4 RNA ligase 2 and T4 DNA ligase) and padlock probes (Target, targeting mRNA ACTB; Random, random padlock probe). (b) The fluorescence intensity of the target RNA-initiated RCA reaction profiled in (a).

Firstly, to demonstrate the feasibility of this principle for RNA detection, we designed a padlock probe targeting synthetic RNA, with the sequence the same as a targeted sequence of mRNA ACTB. The padlock probe and RCA method for target RNA detection is based on RNA-templated ligation. This ligation process is traditionally performed with T4 DNA ligase,^27,28^ and more recently T4 RNA ligase 2,^29^ and has been used for both mRNA^14,22^ and miRNA detection.^29,30^ However, the T4 enzymes are short of catalyzing ligation in the RNA template, thus reverse transcription was adapted for transforming the target RNA to cDNA to circumvent the limitation.^14^ Thus the efficient RNA detection necessitates a highly active RNA-templated ligase. Recently a Chlorella virus DNA ligase now named Splint R was reported with orders of magnitude higher efficiency for the catalyzing ligation of RNA-splinted DNA.^21^ Herein, the target RNA-initiated RCA was conducted using three different ligases, Splint R, T4 RNA ligase 2, and T4 DNA ligase, and a random padlock probe was used as the control. The RCA amplicons with hundreds of copies of the padlock probe tended to form partial double-strand DNA secondary structures, which can be detected using the intercalating dye Sybr Green I. As shown in Fig. 1, the fluorescence intensity for RCA amplicons using Splint R is 1.8 times that of T4 RNA ligase 2, and 1.4 times that of T4 DNA ligase, while the fluorescence signals using a random padlock probe all remained low when using these different ligases. The result was further confirmed by gel electrophoresis. The resulting electrophoresis illustrates that the amount of RCA amplicons was almost doubled using Splint R compared to using T4 RNA ligase 2 or T4 DNA ligase (Fig. S2 in the ESI†). This indicates that the high catalysis activity of Splint R remarkably facilitated the improvement of RNA detection efficiency. The detection efficiency using T4 RNA ligase 2 was lower than for T4 DNA ligase, which is in agreement with the previous report.^29^ Thus, the *in vitro* testing indicates the potential of utilizing Splint R for highly efficient mRNA detection in single cells.

### Evaluation of the ability for imaging of target mRNA in single cells

**Fig. 2:**
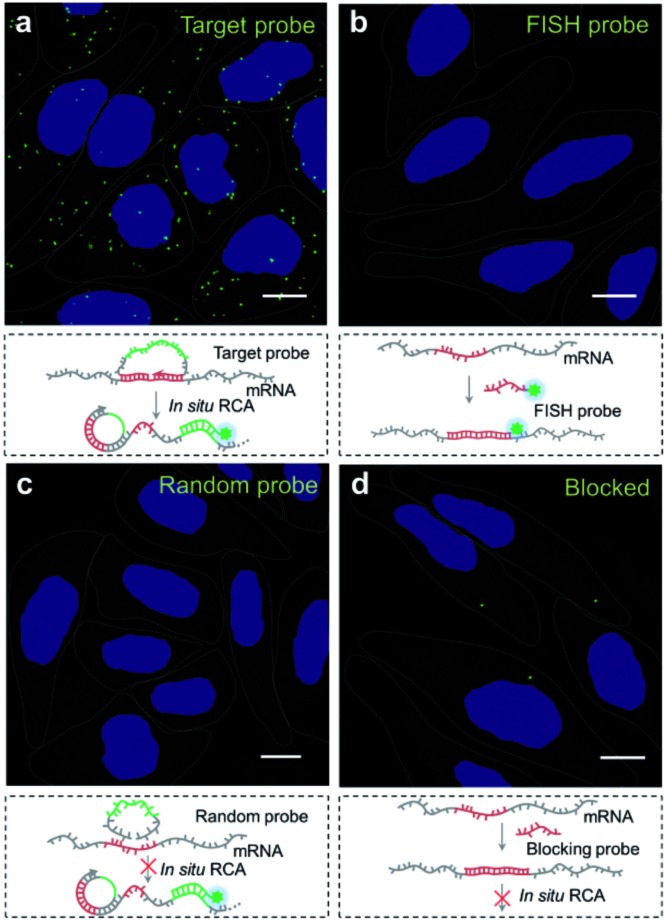
Evaluation of the target RNA-initiated RCA method for imaging of target mRNA in single cells. (a) Imaging of mRNA TK1 using RCA with the target padlock probe; (b) imaging of mRNA TK1 using fluorescence *in situ* hybridization (FISH); (c) imaging of mRNA TK1 using RCA with a random padlock probe; (d) imaging of mRNA TK1 using RCA after blocking the target site of mRNA TK1. The cell nuclei are shown in blue, the RCA amplicons appear as green spots, and the cell outlines are marked by a dotted line. Scale bars: 10 μm.

We then explored the potential of the target RNA-initiated RCA method for the imaging of mRNA in breast cancer cell line MCF-7 cells. A padlock probe was designed for targeting mRNA TK1, which is associated with cell division and was proposed to be a biomarker for breast cancer.^31,32^ As illustrated in Fig. 2a, there were generated superbright dots distinguished from the background inside cells by *in situ* RCA. These superbright dot signals resulted from one-target-one-amplicon *in situ* amplification, thus presumably corresponding to separated mRNAs. Fluorescence images revealed that individual bright fluorescent spots were mostly located randomly in cells with no obvious aggregation seen.

To verify the superbright dot signal coming from the target mRNA by the RCA process, fluorescence *in situ* hybridization (FISH) was performed as a comparison. After the MCF-7 cells were fixed, an Alexa488-labelled FISH probe was *in situ* hybridized with the same sequence of mRNA targeted by the padlock probe, followed with strictly washing to remove the unbound FISH probe. No fluorescence signal distinguished from background was observed (Fig. 2b), which confirmed that the superbright dot resulted from *in situ* RCA. Additionally, to confirm that the superbright dots resulted from the target mRNA TK1, a control experiment was conducted using a random padlock probe. In this case, there was no distinct fluorescence signal (Fig. 2c). Furthermore, we utilized an unlabelled probe to hybridize with the target site of mRNA TK1 prior to carrying out RCA as a blocking process to stop the padlock probe from binding to the target sequence. Only less than one RCA amplicon per cell could be seen (117 cells checked, Fig. 2d), suggesting that the bright dots came from the target mRNA TK1.

Besides, to confirm the accuracy of the method for the spatial mapping of the target mRNAs, we performed a colocalization experiment in which the mRNA TK1 was targeted at two sites. Two padlock probes were designed targeting the region in the middle and the 3′ terminal near poly(A) of mRNA TK1, respectively (Fig. S5a and b†). For the padlock probe targeting the 3′ terminal near poly(A) of mRNA TK1, according to the previous report,^16^ the 3′-5′ RNase activity of phi29 DNA polymerase could remove the poly(A) and convert the target mRNA into a primer to initiate the RCA process. For the padlock probe targeting the middle of mRNA TK1, an extra primer was used to perform the RCA process. The spots in each channel were identified by applying a standard thresholding method for smFISH data analysis (the method is detailed in the Experimental section).^33^ The spots colocalized well in the two different channels, and the distance deviation of spots between the two channels was much less than the size of spots (Fig. S5c†). This confirms that the fluorescent spots were amplified from the target mRNAs and could precisely indicate the location of target mRNAs. The colocalization also implied that the RCA amplicons derived from an extra primer would not diffuse away from the local position of target mRNA which may be because of the crowded environment inside cells.

### Evaluation of the efficiency of mRNA detection using different ligases

**Fig. 3:**
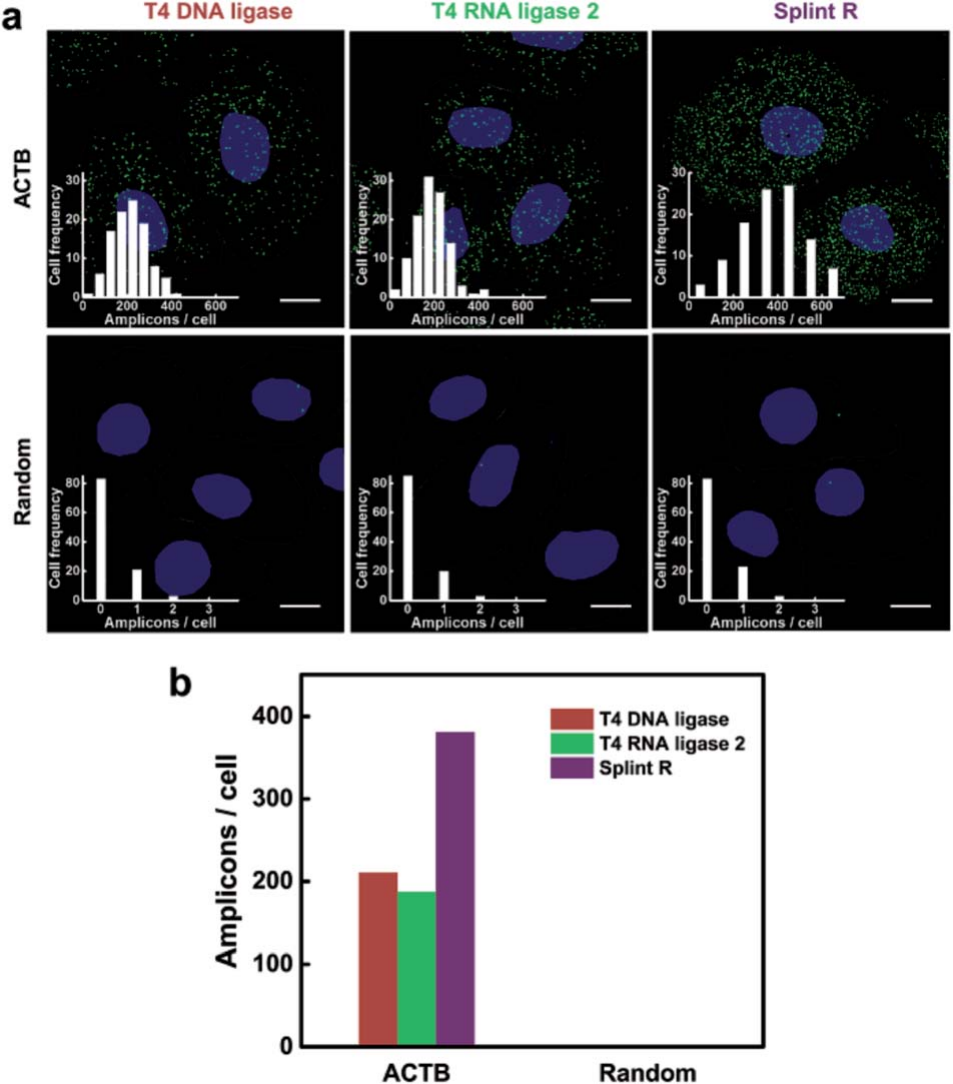
Effect of different ligases on the efficiency of mRNA imaging in single cells. (a) Imaging of mRNA ACTB using target RNA-initiated RCA in the MCF-7 cells with different ligases: T4 DNA ligase, T4 RNA ligase 2 and Splint R. *In situ* RCA was conducted using the padlock probe targeting ACTB and a random padlock probe as control. The cell nuclei are shown in blue, the RCA amplicons appear as green spots, and the cell outlines are marked by a dotted line. Scale bars: 10 μm. Inset: frequency histogram of RCA amplicons per cell detected. (b) Quantification of the average number of RCA amplicons per cell detected in (a).

The performance of the target RNA-initiated RCA method for mRNA detection and imaging is highly dependent on the catalytic activity of the ligase. We chose a highly expressed housekeeping mRNA ACTB to test the detection efficiency of the method. The detection process was carried out using different ligases, T4 DNA ligase, T4 RNA ligase 2 and Splint R. As shown in Fig. 3a, there were generated superbright dots corresponding to RCA amplicons inside cells using all three different ligases. The average number of RCA amplicons per cell detected using T4 DNA ligase, T4 RNA ligase 2 and Splint R was 210.51, 187.18 and 380.71, respectively (Fig. 3b). While using the random padlock probe, there were less than 0.3 RCA amplicons per cell using all three ligases, confirming that the RCA amplicons were amplified from the target mRNA ACTB. The detection efficiency for targeting mRNA ACTB using T4 DNA ligase, T4 RNA ligase 2 and Splint R was 10.9%, 9.7% and 19.7%, respectively, on the basis of a comparison with quantitative reverse transcription PCR (RT-qPCR) data. The detection efficiency using Splint R for moderately expressed mRNA TK1 was over 20% (Table S3 in the ESI†). The detection efficiency of the *in situ* RCA method was significantly improved by the utilization of Splint R. The efficient catalytic activity of ligation of Splint R on the RNA template greatly improved the mRNA detection efficiency, which was consistent with the *in vitro* test. The detection efficiency could be further improved by optimizing the padlock probe sequence and targeting a more accessible binding site of mRNA (discussed in the ESI†).^17^ As the mRNA has a long sequence and complex secondary structures which could not be directly used as a primer for RCA,^22^ we performed the *in situ* RCA detection process for mRNA detection using an extra short DNA sequence as the primer. When the *in situ* RCA process was conducted without a primer, the number of amplicons in the cells was sharply cut down (Fig. S6†). This confirmed the necessity of the primer to initiate the RCA process. The detection efficiency for mRNA in earlier papers was only 1% or 4–7%.^16,22^ In this work, by using a more efficient ligase and an extra primer, the direct detection of mRNA using padlock probes was developed to be of a comparable performance to the method that relied on reverse transcription (with a detection efficiency of 15%),^14^ with much simplicity and easy operability.

### Demonstration of the specificity for mRNA imaging in single cells

**Fig. 4:**
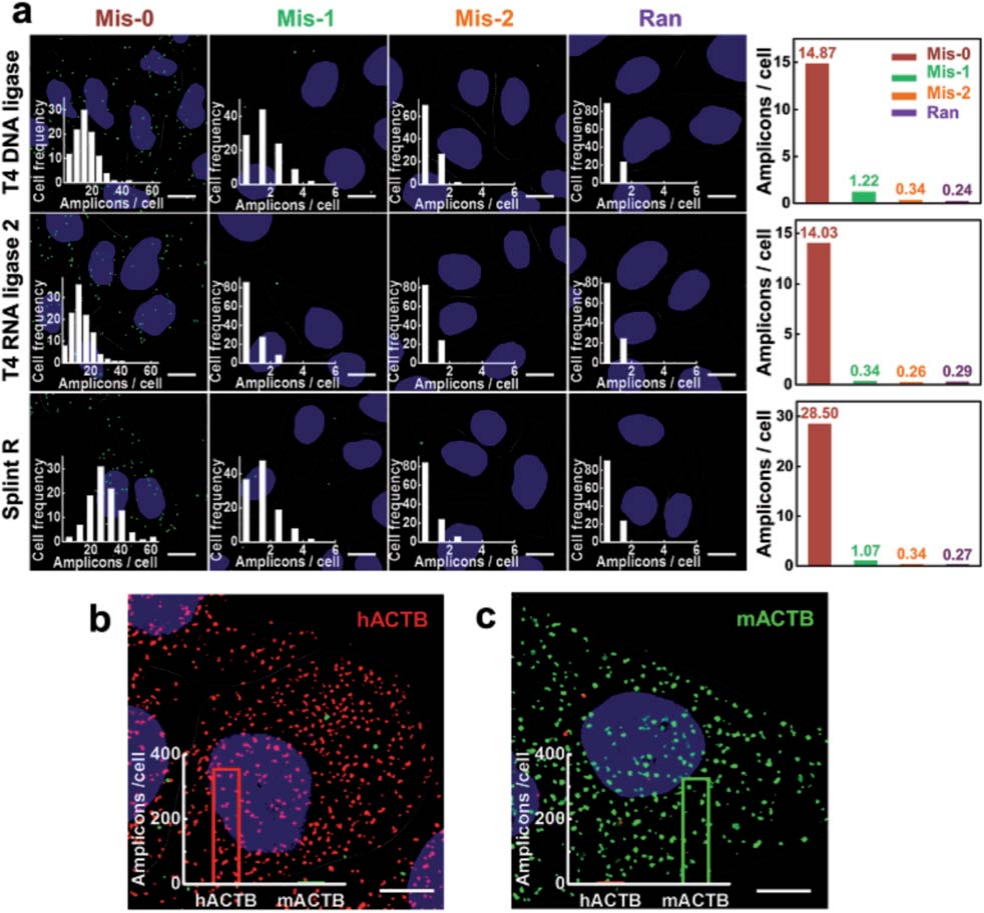
Demonstration of the specificity for mRNA imaging in single cells. (a) The RCA reactions were carried using T4 DNA ligase, T4 RNA ligase 2 and Splint R. Four padlock probes were designed for target mRNA TK1: one perfectly matching with the target sequence of mRNA TK1 (Mis-0), two probes with one (Mis-1) or two (Mis-2) mismatching bases compared to target mRNA TK1 and one random probe (Ran). Inset: frequency histogram of RCA amplicons per cell detected. The right column is the average number of RCA amplicons detected in MCF-7 cells using the padlock probes Mis-0, Mis-1, Mis-2 and Ran; (b and c) detection of a single nucleotide difference in mRNA ACTB in human MCF-7 cells (b) and mouse 4T1 cells (c). Inset: the quantification of the average number of RCA amplicons detected (100 cells counted). The cell nuclei are shown in blue, the RCA amplicons appear as green or red spots, and the cell outlines are marked by a dotted line. Scale bars: 10 μm.

The recognition process for target sequences based on the enzyme-catalyzed ligation process gives the padlock probe and RCA based method high specificity. The specificity of target RNA-initiated RCA using different ligases was further tested. Four padlock probes were designed for target mRNA TK1: one perfectly matching with the target sequence of mRNA TK1 (Mis-0), two probes with one (Mis-1) or two (Mis-2) mismatched bases compared to the target mRNA TK1 and one random probe (Ran). When introducing a one- or two-base mismatch, the number of RCA amplicons per cell dramatically decreased when using all three ligases (Fig. 4). The average number of RCA amplicons per cell with the Mis-0 padlock probe using T4 DNA ligase, T4 RNA ligase 2 and Splint R was 14.87, 14.03 and 28.50, respectively, while when using Mis-1 and Mis-2 padlock probes there was less than 1.5 per cell and 0.4 per cell, respectively, much less than when using the perfectly matching padlock probe Mis-0. The discrimination factor between the perfect match and one-base mismatch was estimated to be 12.19, 41.26 and 26.63 using T4 DNA ligase, T4 RNA ligase 2 and Splint R, respectively. Among these three ligases, the highest selectivity was obtained using T4 RNA ligase 2. This is consistent with the previous report that T4 RNA ligase 2 outperformed other ligases in the specificity of RNA detection.^29^ Nevertheless, the target RNA-initiated RCA method could also be sufficient to discriminate one-nucleotide variations with the utilization of Splint R, with much higher detection efficiency compared to T4 DNA ligase or T4 RNA ligase 2.

To further demonstrate its high selectivity, the method with the utility of Splint R was exploited to genotype mRNAs with single-nucleotide variants. We used a single-nucleotide difference between the human and mouse ACTB sequences as the genotyping target. Two padlock probes were designed to target the human and mouse ACTB, respectively. The two padlock probes were both added to the two fixed cell lines, human MCF-7 cells and mouse 4T1 cells to image the target ACTB. Abundant RCA amplicons (both over 300 amplicons per cell) were generated in the corresponding cells, while the non-specific signals were rarely presented (about 3 amplicons per cells) (Fig. 4b and c). This confirms that the method could gain the specificity for genotyping mRNA in single cells with near-single-molecule resolution.

### Mapping the spatial distribution of mRNAs in single cells

**Fig. 5:**
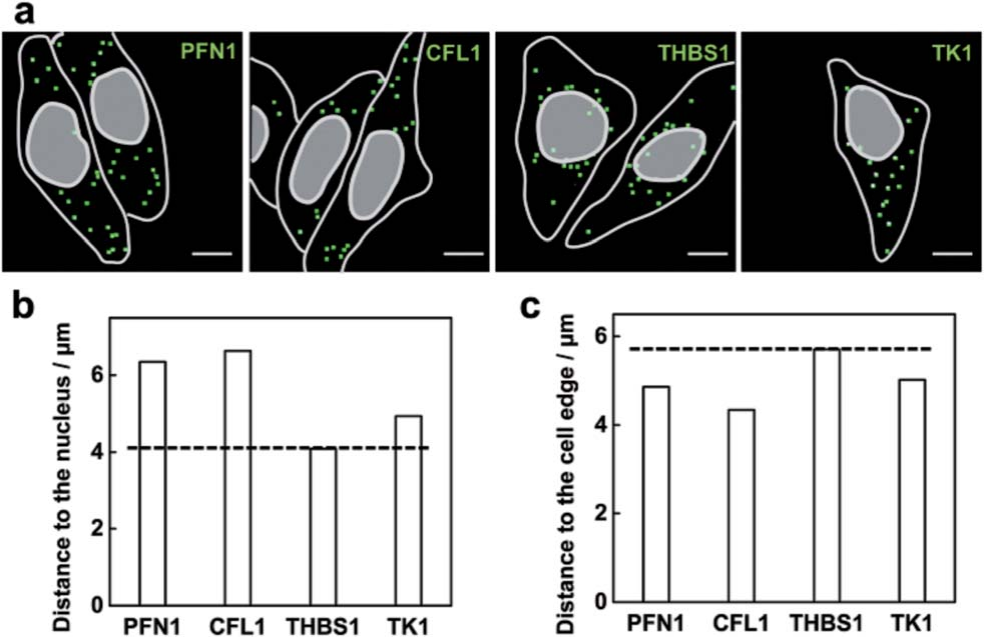
Mapping the spatial distribution of mRNAs in the MCF-7 cells. (a) Example images of the spatial distributions observed for the mRNAs PFN1, CFL1, THBS1 and TK1. The RCA amplicons are marked with green squares, and the outlines of cell and nucleus are marked by a gray line. Scale bars: 10 μm. The quantification of average distances for the four mRNA species to the nucleus (b) and the cell edge (c).

The method capable of locating mRNAs allows us to investigate the spatial distribution of mRNAs. As a proof of principle, four mRNAs TK1, PFN1, CFL1 and THBS1 were chosen to be imaged. From the fluorescence images of each mRNA detected (Fig. 5a), these different mRNA species appeared with different distribution patterns in the cells. The mRNA THBS1 was mostly located in the perinuclear region, the mRNAs PFN1 and CFL1 appeared enriched near the cell periphery, and the mRNA TK1 appeared with no bias neither close to the nucleus or the cell edge. Quantitative analysis of the distances between each mRNA and the cell nucleus or the cell periphery further confirmed the visual impression. The average distance to the nucleus for the mRNAs PFN1, CFL1, and TK1 was 1.55, 1.62 and 1.21 times that for the mRNA THBS1, respectively (Fig. 5b). The average distance to the cell edge for the mRNA PFN1, CFL1, and TK1 was 0.85, 0.76 and 0.88 times that for the mRNA THBS1, respectively (Fig. 5c). The different spatial patterns of the mRNAs may imply their different functions. The THBS1 gene is identified to encode extracellular proteins and the extracellular proteins must pass through the secretion pathway, which requires the translation of the mRNA at the endoplasmic reticulum that is located around the nucleus.^8^ Thus the mRNA THBS1 tends to be located in the perinuclear region. This result is consistent with a previous report on a spatial study of the mRNA THBS1 by smFISH.^8^ mRNAs that encode proteins related to cytoskeleton were reported enriched near the cell periphery.^34^ PFN1 and CFL1 are genes that encode the actin-binding proteins.^35^ Abundant amounts mRNAs PFN1 and CFL1 appeared near the cell periphery. The ability for the spatial mapping of mRNAs in single cells may offer us new ways to predict and study the functions of mRNAs.

### Multiplex imaging of mRNAs in single cells

**Fig. 6:**
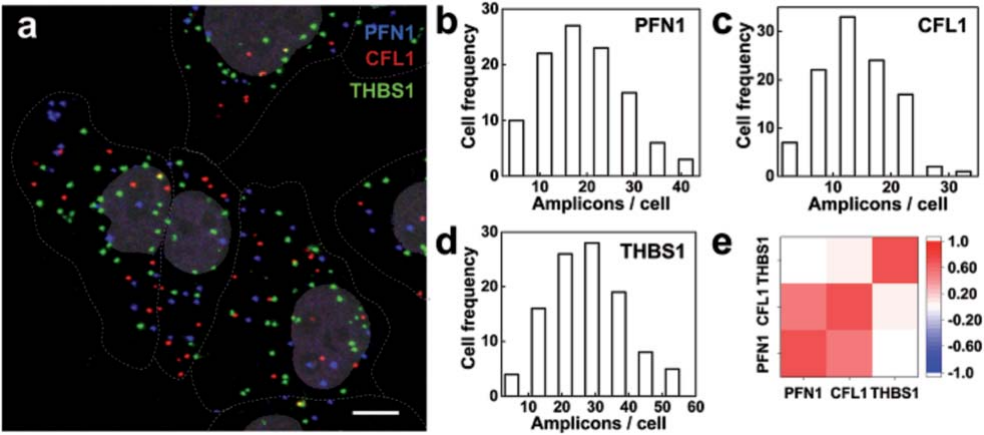
Multiplex imaging of mRNAs in single cells. (a) Fluorescence image of the three target mRNAs PFN1, CFL1 and THBS1 simultaneously visualized using target RNA-initiated RCA. The cell nuclei are shown in gray and the cell outlines are marked by a dotted line. Scale bars: 10 μm; (b–d) histograms of the per cell detected number of RCA amplicons corresponding to the three target mRNAs in the MCF-7 cells. (e) Matrix of the pairwise correlation coefficients of the cell-to-cell variation in expression for the three measured target mRNAs.

We then explored the multiplex ability for simultaneously imaging different mRNA species. Three mRNAs PFN1, CFL1 and THBS1 were targeted and the RCA amplicons were hybridized with different detection probes labeled as Alexa555, Cy5 and Alexa488, respectively. The three mRNA species were detected in different fluorescence channels (Fig. 6a). The mRNAs PFN1, CFL1 and THBS1 simultaneously detected similar spatial patterns compared with those detected alone. The ability for the parallel profiling of multiple mRNA species in single cells enabled the investigation on the gene expression heterogeneity and correlation. As shown in the frequency histogram of RCA amplicons per cell (Fig. 6b–d), there was large cell-to-cell variation in gene expression in the MCF-7 cell lines. For example, the number of RCA amplicons for the mRNA THBS1 ranged from 4 to 54 per cell (the average was 26.72 per cell). This variability suggests that even genetically identical cells would exhibit significant cell-to-cell variation in gene expression.^3,36^ At the single-cell level, we could take advantage of the natural stochastic fluctuations in gene expression to analyze whether the transcriptional process of different genes is coordinated.^8^ The expression correlation analysis indicates that the expression of PFL1 and CFL1 was correlative in the MCF-7 cells (Fig. 6e). The two genes encode the actin-binding proteins profilin 1 and cofilin 1, respectively, and both are involved in the assembly process of the cytoskeleton.^35^ Analysis of covariations in the expression levels of different genes on the single-cell level could reveal coregulated genes and help to elucidate gene regulatory pathways and mechanisms.

## Conclusions

In summary, we have built a robust method for the direct detection of mRNA, enabling the imaging of mRNA at single-nucleotide and near-single-molecule resolution in single cells. In contrast to previous reports, the proposed method can achieve the efficient detection of mRNA without reverse transcription by using a Splint R ligase which has a high ligation catalytic activity on the RNA template. Benefiting from the ligation-based recognition process, the method is highly specific and can distinguish between mRNA sequences with single-nucleotide variations. Single-cell mRNA imaging data show that mRNAs with different functions may be represented by distinct spatial patterns, and genes with relevant functions may express coordinately. We believe that our method to visualize mRNAs with high spatial resolution and sequence specificity in single cells, potentially capable of profiling gene expression, single-nucleotide variants and mRNA alternative splicing at single-cell level, will be of great value for both basic research and clinical diagnosis.

## Experimental section

### Proteins, reagents and DNA sequences

T4 polynucleotide kinase, T4 DNA ligase, phi29 DNA polymerase, RiboLock RNase Inhibitor, RevertAid First Strand cDNA Synthesis Kit and SYBR select master mix were bought from Thermo Fisher Scientific (Waltham, USA). T4 RNA ligase 2, Splint R and HiScribe™ T7 High Yield RNA Synthesis Kit were purchased from New England Biolabs (Beijing, China). Deoxyribonucleotide mixtures (dNTPs) were bought from Beijing DingGuo Biotechnology Co., Ltd. (Beijing, China). The salmon sperm DNA, 20× SSC buffer (pH 7.4) and 4% paraformaldehyde in PBS buffer were purchased from Beijing Solarbio Science & Technology Co., Ltd. (Beijing, China). TransZol and TransScript one-step gDNA removal and cDNA synthesis were bought from Transgen biotech Co., Ltd. (Beijing, China). Diethyl pyrocarbonate (DEPC), formamide, Tween-20 and Triton-X100 were purchased from Sigma-Aldrich (St. Louis, USA). All of the solution and deionized water used were treated with DEPC and autoclaved to be protected from RNase degradation.

The DNA sequences (Table S1 in the ESI†) were purchased from Shanghai Sangon Biological Engineering Technology & Services Co., Ltd (Shanghai, China). The FISH probe and the RCA detection probe were modified with Alexa488, Alexa555 and Cy5, and were bought from Thermo fisher Scientific, all were HPLC purified. RNA sequences were created by *in vitro* T7 transcription reactions with ordered single stranded DNAs used as templates.

### *In vitro* target RNA-initiated RCA reaction

The linear padlock probe was first phosphorylated at a volume of 20 μL produced by adding 2 μL of the linear padlock probe (100 μM), 2 μL of 10× T4 polynucleotide kinase reaction buffer, 15.5 μL of DEPC-treated H_2_O and 0.5 μL of T4 polynucleotide kinase (10 U μL^–1^). The ligation reaction was conducted at a volume of 40 μL produced by adding 4 μL of the phosphorylated padlock probe (100 nM), 4 μL of 10× ligase reaction buffer, 4 μL of target RNA solution (1 μM), 27.5 μL of DEPC-treated H_2_O and 0.5 μL of ligase (T4 DNA ligase (5 U μL^–1^), T4 RNA ligase 2 (10 U μL^–1^) or Splint R (25 U μL^–1^)). Before the ligase was added, the reaction mixture was heated at 55 °C for 5 min. After the reaction mixture had been annealed at 39 °C for 30 min and cooled to room temperature, the ligase was added and the ligation reaction was conducted at 30 °C for 30 min. The product of the ligation reaction was added to 10 μL of the RCA reaction mixture containing 5 μL of 10× phi29 DNA polymerase reaction buffer, 4 μL of dNTPs (10 mM for each of dATP, dGTP, dCTP and dTTP), 0.5 μL of DEPC-treated H_2_O and 0.5 μL of phi29 DNA polymerase (10 U μL^–1^). The RCA reaction was performed at 37 °C for 2 h and terminated by incubation at 65 °C for 10 min. The RCA products were then mixed with 100× Sybr Green I to become the final solution with 0.5× Sybr Green I. The fluorescence spectra were measured using EnVision Multilabel Plate Readers (PerkinElmer, USA). The excitation wavelength was 494 nm, and the fluorescence emission spectra were recorded from 513 nm to 700 nm.

### Sample pretreatment for imaging

Cells were seeded on a 22 mm × 22 mm gelatin coated coverglass (VWR, USA) enclosed by a PDMS with a chamber (5 mm in diameter) and allowed to attach. When the cells reached the desired confluency, they were fixed in 4% (w/v) paraformaldehyde in 1× phosphate buffered saline (PBS) for 15 min at room temperature (20–25 °C), and then washed twice with 1× DEPC-treated PBS (DEPC-PBS). After fixation, the cells were permeabilized for 5 min with 0.5% v/v Triton-X100 in 1× PBS at room temperature. This was followed by two brief washes with DEPC-PBS.

### Imaging of mRNAs in single cells by target RNA-initiated RCA

The hybridization of the padlock probe with the target mRNA was conducted in a 20 μL mixture containing 2 μL of each phosphorylated padlock probe (10 μM), 2 μL of yeast tRNA (10 mg mL^–1^), 1 μL of DTT (100 mM) and 0.5 μL of RiboLock RNase Inhibitor (40 U μL^–1^) overnight at 37 °C. The sample was washed using PBS-T (DEPC-PBS with 0.05% Tween-20) twice for 3 min at room temperature. A 10 μL circularization reaction mixture (1 μL of 10× ligase reaction buffer, 1 μL of ligase (T4 DNA ligase (5 U μL^–1^), T4 RNA ligase 2 (10 U μL^–1^) or Splint R (25 U μL^–1^)), 0.25 μL of RiboLock RNase Inhibitor (40 U μL^–1^), and 6.75 μL of DEPC-treated H_2_O) was then added to the sample and incubated at 37 °C for 2 h. After circularization, the sample was incubated with a 20 μL mixture containing 1 μL of primer (4 μM), 2 μL of 20× SSC, 2 μL of formamide, 1 μL of DTT (100 mM), 13.5 μL of DEPC-treated H_2_O and 0.5 μL of RiboLock RNase Inhibitor (40 U μL^–1^) for 60 min at 37 °C, following a wash using PBS-T. RCA was then performed at a volume of 10 μL containing 1 μL of 10× phi29 DNA polymerase reaction buffer, 3 μL of dNTPs (10 mM of each of dATP, dGTP, dCTP and dTTP), 5.25 μL of DEPC-treated H_2_O, 0.5 μL of phi29 DNA polymerase and 0.25 μL of RiboLock RNase Inhibitor (40 U μL^–1^). The reaction mixture was incubated for 120 min at 37 °C. The incubation was followed by a wash in PBS-T. RCA amplicons were visualized by being hybridized with 100 nM fluorophore-labelled detection probes in 2× SSC, 10 ng μL^–1^ salmon sperm DNA and 15% formamide at 37 °C for 30 min. The incubation was followed by three washes in PBS-T. The slides were ready for imaging after being mounted with Fluoromount-G (containing 4′,6-diamidino-2-phenylindole (DAPI) to counterstain the cell nuclei).

### Image acquisition and analysis

Fluorescence imaging was performed using a Leica TCS SP5 inverted confocal microscope (Leica, Germany). The images of RCA amplicons were acquired using a 40× oil-immersion objective. An Ar^+^ laser (488 nm) was used as an excitation source for the Alexa488-labelled probe, and a 500–535 nm bandpass filter was used for fluorescence detection. The Alexa555 dye was excited with a HeNe543 (543 nm) laser and detected with a 555–620 nm bandpass filter. The Cy5 dye was excited with a HeNe633 (633 nm) laser and detected with a 650–750 bandpass filter. The DAPI dye was excited with a 405 nm laser line and detected with a 430–550 nm bandpass filter. Images were collected as z-stacks to ensure that all RCA amplicons were imaged. Stacks of images were taken with 0.2 μm between the z-slices, and combined to a single image by maximum intensity projection (MIP) using LAS AF Version 2.6.3.8173 software. The stitched image in different channels with a size of 164 μm × 164 μm and pixels of 160 nm × 160 nm was used for further image analysis.

The superbright spot in the fluorescence image supposed to be a single RCA amplicon was distinguished from the background signal by thresholding the images using ImageJ version 1.46r software. The threshold value was chosen based on a previously published smFISH thresholding method,^33^ which lay in a region of the plateau over which the number of spots detected was insensitive to the threshold chosen (Fig. S3†). The nucleus was also identified by thresholding the images in the DAPI channel. The outline of the cell was marked out from the bright field images. The location and size of RCA amplicons, nucleus and cells could be used for further analysis. The number of RCA amplicons in a single cell was determined by counting the isolated fluorescent spots inside the outline of the cell using ImageJ software (Fig. S4†). The distances for RCA amplicons (supposed corresponding to separated mRNAs) to the nucleus and the cell edge were obtained using the flood algorithm by Python 2.7.13.
