# Supplementary material for: Highly specific imaging of mRNA in single cells by target RNA-initiated rolling circle amplification
Source: Chem Sci. 2017 Mar 7;8(5):3668–75. doi: 10.1039/c7sc00292k (PMC5437493; doi:10.1039/c7sc00292k)
Supplement: Supplementary file 1 [file SC-008-C7SC00292K-s001.pdf]

Electronic Supplementary Material (ESI) for Chemical Science.
This journal is © The Royal Society of Chemistry 2017

Highly specific imaging of mRNA in single cells by target RNA-initiated rolling circle amplification

*Ruijie Deng, Kaixiang Zhang, Yupeng Sun, Xiaojun Ren and Jinghong Li**

Department of Chemistry, Key Laboratory of Bioorganic Phosphorus Chemistry & Chemical Biology, Beijing Key Laboratory for Microanalytical Methods and Instrumentation, Tsinghua University, Beijing 100084, China

* To whom correspondence should be addressed. Email: jhli@mail.tsinghua.edu.cn

Table of contents

1. SI Materials and methods S2
2. Table S1 Oligonucleotide sequences S4
3. Table S2. Major specifications of target RNA-initiated RCA in comparison with other RCA-based RNA imaging methods S6
4. Fig. S1. Comparing the methods for single-cell mRNA imaging with or without reverse transcription S6
5. Fig. S2. Electrophoresis analysis of target RNA-initiated RCA products using different ligases S7
6. Fig. S3. Identification of RCA amplicons in the fluorescence image S8
7. Fig. S4. Schematic illustration for quantifying and spatial mapping target mRNAs in single cells S9
8. Fig. S5 Co-localization of mRNAs targeted by different sites S10
9. Fig. S6. The effect of the primer on the mRNA detection efficiency. S11
10. The detection efficiency of in situ amplification and possible improvement S12
11. Fig. S7. Expression analysis of target mRNAs in the MCF-7 cells by RT-qPCR. S13
12. Fig. S8. Histograms of the distances for the mRNAs PFN1, CFL1, THBS1 and TK1 to the nucleus and the cell edge of each cells S14
13. Table S3. The average numbers of ACTB and TK1 in the MCF-7 cells measured by target RNA-initiated RCA and RT-qPCR S15
14. References S15

SI Materials and methods

Electrophoresis analysis for RCA amplicons

The RCA amplicons in 6 μl 1×gel loading buffer were used for gel electrophoresis. Agarose was prepared with 40 mM Tris-acetate running buffer containing 40 mM Tris, 20 mM acetic acid, 2 mM EDTA (TAE, pH 8.0) to form 1% agarose-TAE sol-gel with 1×Unired dye. The gel electrophoresis was performed on the prepared gel in TAE at 100 V for 90 min. After electrophoresis, the gel was visualized via ChampGel 5000 (Beijing Sage Creation Science Co., Ltd, China).

Cell culture

The MCF-7 and 4T1cell line was kindly provided by the National Center for Nanoscience and Technology (Beijing, China). The 4T1 cells were cultured in a RPMI 1640 medium supplemented with 15% fetal calf serum, 100 μg mL^{-1} of streptomycin, and 100 units mL^{-1} of penicillin. The MCF-7 cells were cultured in a DMEM medium supplemented with 15% fetal calf serum, 100 μg mL^{-1} of streptomycin, 100 units mL^{-1} of penicillin and 0.01 mg/ml human recombinant insulin. Cells were all cultured at 37 °C in a humidified incubator containing 5% CO_2.

In situ imaging of mRNA by FISH

Cells were seeded on a 22 mm × 22 mm gelatin coated coverglass (VWR, Radnor, USA) enclosed by a PDMS with a chamber (5 mm in diameter) and allowed to attach. When the cells reached the desired confluency, they were fixed in 4% (w/v) paraformaldehyde in 1 × phosphate buffered saline (PBS) for 15 min at room temperature (20-25 °C), washed twice with 1 × DEPC-treated PBS (DEPC-PBS). After fixation, the cells were permeabilized for 5 min with 0.5% v/v Triton-X100 in 1 × PBS at room temperature. This was followed by two brief washes with DEPC-PBS. The hybridization of FISH probe with the target mRNA TK1 was conducted in a 20 μL mixture containing 100 nM Alexa488-labelled DP-FISH-TK1 probe in 2 × SSC, 10 ng/μL salmon sperm DNA and 15% formamide at 37 °C for 12 h. The incubation was followed by three washes in PBS-T. The slides were ready for imaging after mounted with Fluoromount-G (with DAPI).

Real-time quantitative PCR (RT-qPCR) analysis of mRNA inside cells

Total RNA was extracted from MCF-7 cells using TransZol following the manufacturer's

instructions. The cDNA samples were prepared using TransScript one-step gDNA removel and cDNA synthesis kit. Briefly, a total volume of 20 μL solution containing 2 μL of the total RNA (50 ng-5 μg), 1 μL anchored oligo$(dT)_{18}$ primer (0.5 μg/μl), 10 μL 2 × TS reaction mix, 1 μL TransScript RT/RI enzyme mix, 1 μL gDNA remover and 5 μL RNase-free water was incubated at 42 °C for 15 min followed by heat inactivation of reverse transcriptase for 5 s at 85 °C. The cDNA samples were stored at -80 °C for future use.

qPCR analysis of mRNA was performed with SYBR select master mix according to the manufacturer's instructions on a Bio-Rad C1000™ (Bio-Rad, USA). The 20 μL reaction solution contained 2 μL of cDNA sample, 10 μL 2 × SYBR Select master mix, 2 μL forward primer (5 μM), 2 μL reverse primer (5 μM), 4 μL RNase-free water. The qPCR conditions were as follows: staying at 50 °C for 2 min for the hot start, annealing at 95 °C for 2 min, then followed by 40 cycles of 95 °C for 15 s and 60 °C for 60 s. C_t values were converted into absolute GAPDH copy numbers using a standard curve from a control RNA (human GAPDH mRNA in RevertAid First Strand cDNA Synthesis Kit). A standard curve was prepared from cDNA solutions corresponding to the serially diluted solutions of human GAPDH mRNA. The volumes and components of reverse transcription and qPCR reaction mixtures were the same as those for the test samples. Obtained results are presented as the copies of mRNA per cell. The experiment was repeated three times. The copy number of target mRNAs ACTB and TK1 was evaluated by referring to the expression of GAPDH mRNA using the $2^{-\Delta\Delta Ct}$ method.[1]

Table S1. Oligonucleotide sequences

Name	Sequences (5'-3')	Description
T-ACTB	TAATACGACTCACTATAGGGGCCGCCTAGAAGCATTTGCGGTGGACGATGG	Used as the templates for transcription to produce the target RNA sequence for *in vitro* testing
P-TK1	CTCCTTCTCTGTGCCAGTGCGAACTACTACTCTCTCTCAATTCTGCTACTGTACT TGCGTCTATTTTCTGGAGCCCCCAATCACCTCGAC	Padlock probes for *in situ* imaging mRNA TK1, The bases marked in red indicate the mismatched bases between the padlock probe and target sequence.
P-TK1-mis1	ATCCTTCTCTGTGCCAGTGCGAACTACTACTCTCTCTCAATTCTGCTACTGTACT TGCGTCTATTTTCTGGAGCCCCCAATCACCTCGAC	
P-TK1-mis2	ATCCTTCTCTGTGCCAGTGCGAACTACTACTCTCTCTCAATTCTGCTACTGTACT TGCGTCTATTTTCTGGAGCCCCCAATCACCTCGAA	
P-TK1-random	ATGCATCCGTTATTAAGTGCGAACTACTACTCTCTCTCAATTCTGCTACTGTACT TGCGTCTATTTTCTGGAGCCTCTACTGTCTTACGTT	
P-TK1-terminal	TTTTCAATTAGTTAATATTTTTTTATTTATTATTTTATTGCGTCTATTTTCTGGA GCCATTATTTTTTTTTTTCCACCAACCAGTGAA	Padlock probes for targeting the 3'-terminal site near poly (A) of mRNA TK1
P-ACTB-C	TGCGGTGGACGATGGTTTTTTTCTCAATTCTGCTACTTTACTACCTCAATTCTGC TACTGTACTACTTTTTTTCCGCCTAGAAGCATT	Padlock probes for *in situ* imaging mRNA ACTB in the MCF-7 cells
P-ACTB-random-C	ATGCATCCGTTATTATTTTTTTCTCAATTCTGCTACTGTACTACCTCAATTCTGC TACTGTACTACTTTTTTTTCTACTGTCTTACGTT	
P-hACTB	TTGCACATGCCGGAGAATTTTTTTATTTATTATTTTATTTGCGTCTATTTTCTGG AGCCATTTTTTCTTTATTCCCGCGAAGCCGGCC	Padlock probes for genotyping mRNA ACTB
P-mACTB	TTGCACATGCCGGAGAATTTTTAACTATACAACATACTACCTCAAACTATACAAC ATACTACCTCATTTTTTTCCCGCGAAGCCGGCT	
P-PFN1	AAATGGTTTGTGTGTTTTTTAGTGCGAACTACTACTCTCTTTTTTTCTCAATTCT GCTACTGTACTACATATAATGGCCCAAAAAAATA	Padlock probes for simultaneously imaging of mRNAs PFN1, CFL1 and THBS1
P-CFL1	GTCAGCTTCTTCTTGTATTTTTTTATTTATTATTTTATTGCGTCTATTTTCTGGA GCCATTATTTTTTTTTTTTCATGCTTGATCCCT	
P-THBS1	GCACAAGGGATGGGGTATTTTTTTATTTATTATTTTATAACTATACAACATACTA CCTCATATTTTTTTTTTTTCTCCCTGGAAATAT	
P-PFN1-S	AAATGGTTTGTGTGTTTTTTAGTGCGAACTACTACTCTCTTTTTTTAACTATACA ACATACTACCTCAATATAATGGCCCAAAAAAATA	Padlock probes for mapping the spatial distributions of mRNAs PFN1, CFL1, THBS1 and TK1
P-CFL1-S	GTCAGCTTCTTCTTGTATTTTGCGTCTATTTTCTGGAGCCATTTTTAACTATACA ACATACTACCTCATTTTTTCATGCTTGATCCCT	

P-THBS1-S	GCACAAGGGATGGGGTATTTTTTTATTTATTATTTTATAACTATACAACATACTACCTCATATTTTTTTTTTTTCTCCCTGGAAATAT	
P-TK1-S	CTCCTTCTCTGTGCCAATTTTTAACTATACAACATACTACCTCAAACTATACAACATACTACCTCATTTTTTTCCCAATCACCTCGAC	
DP-488-1	Alexa488-AACTATACAACATACTACCTCA	Fluorophore-labelled detection probes for visualizing RCA amplicons
DP-488-2	Alexa488-AGTGCGAACTACTACTCTCT	
DP-555	Alexa555-CTCAATTCTGCTACTGTACTAC	
DP-Cy5	Cy5-CTCAATTCTGCTACTGTACTAC	
DP-FISH-TK1	Alexa488-GGCACAGAGAAGGAGGTCGAGGTGATTGGG	Fluorophore-labelled detection probe for visualizing mRNA TK1 by FISH
Blocking probe	GGCACAGAGAAGGAGGTCGAGGTGATTGGG	To block the binding site of mRNA TK1 for RCA detection
Primer	AGTACAGTAGCAGAATTGAG	Used as the primer for RCA

Table S2. Major specifications of target RNA-initiated RCA in comparison with other RCA-based RNA imaging methods

Methods	Target RNA/DNA	Reverse transcription	Detection efficiency	Reference
Target RNA-initiated RCA	**mRNA**	**No**	**＞20%**	**This work**
Target-primed RCA with padlock probes	mRNA	Yes	~15%	*Nat Methods 2010, 5, 395-397; Nat Methods 2013, 10, 857-860*
Target RNA-primed RCA	mRNA	No	~1%	*RNA 2009, 15, 765-771*
RNase III-assisted target RNA-primed RCA	mRNA	No	~4-7%	*RNA 2010, 16, 1508-1515*
Fluorescent *in situ* sequencing (FISSEQ)	mRNA/ncRNA/rRNA	Yes	＜1%	*Science 2014, 343, 1360-1363*

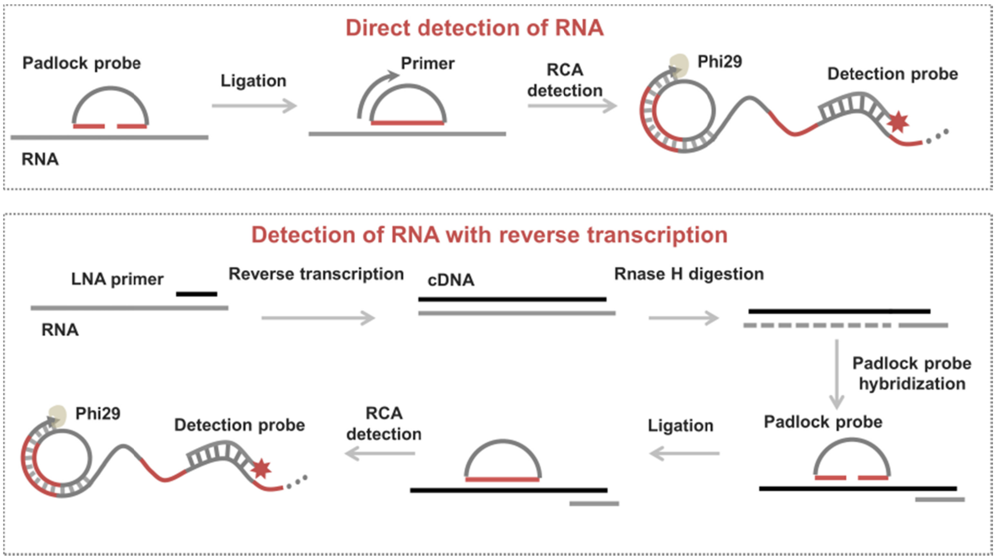

Fig. S1. Comparing the methods for single-cell mRNA imaging with or without reverse transcription

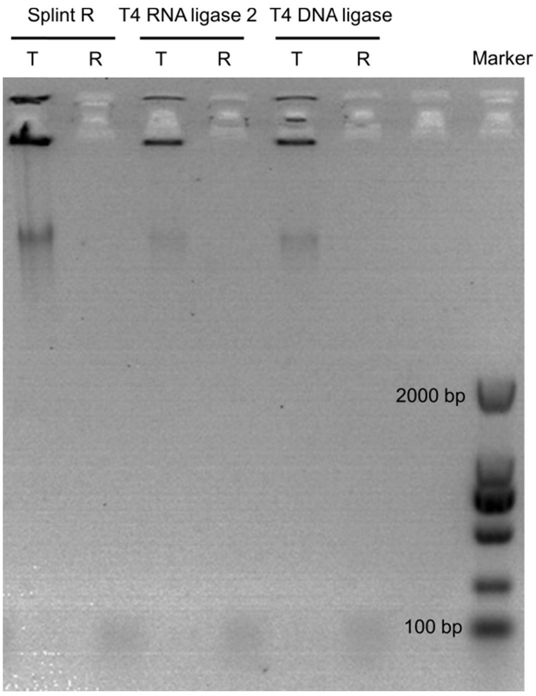

Fig. S2. Electrophoresis analysis of target RNA-initiated RCA products using different ligases. The target RNA-initiated RCA reaction was carried out using different ligases (Splint R, T4 RNA ligase 2 and T4 DNA ligase) and padlock probes (T, padlock probe for targeting mRNA ACTB; R, random padlock probe).

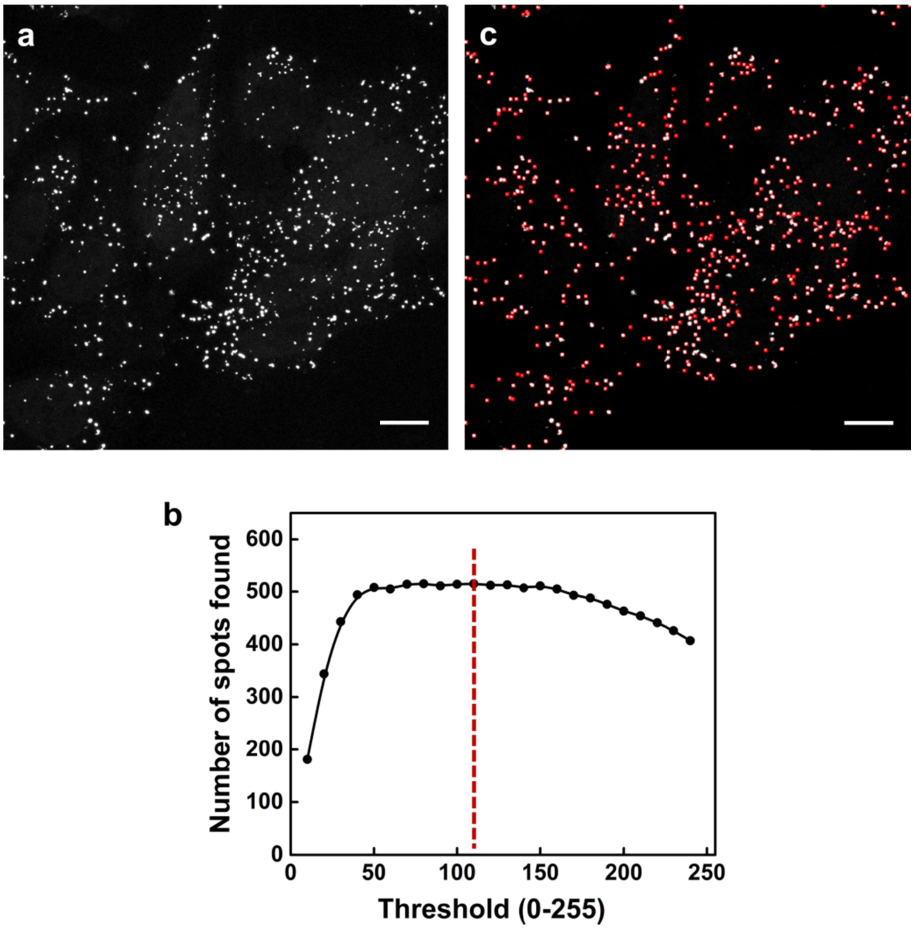

Fig. S3. Identification of RCA amplicons in the fluorescence image. (a) Raw fluorescence image obtained (by maximum intensity projection) from imaging mRNA TK1 in the MCF-7 cells. (b) Plots of the number of RCA amplicon spots found upon setting a threshold for the image from (a) against the threshold value, ranging from 0 to the maximum intensity of the image (0 to 255 for 8 bit image). There is a region of plateau over which the number of spots detected is insensitive to the threshold chosen. The red dashed line represents the threshold used (within the plateau) for determining the number of RCA amplicon in the image. (c) Image showing the results of using the threshold represented by the red dashed line in (b) on the image in (a), with each identified spot marked with a red square. The spots detected in (c) correspond very well with those in (a). Scale bar: 20 μm.

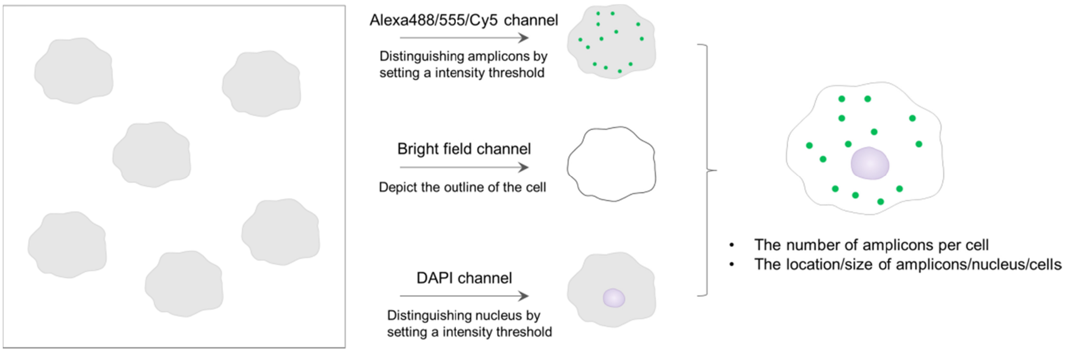

Fig. S4. Schematic illustration for quantifying and spatial mapping target mRNAs in single cells. The RCA amplicons were identified in the fluorescence images in the channel (Alexa555, Cy5 or Alexa488) of detection probe by setting the intensity threshold. The nucleus was also identified by thresholding the images in the DAPI channel. The outline of the cell was marked out from the bright field images manually. The number of RCA amplicons in single cell was determined by counting the isolated fluorescence spots inside the outline of the cell. The spatial information of RCA amplicons, nuclei and cells could be used for further analysis.

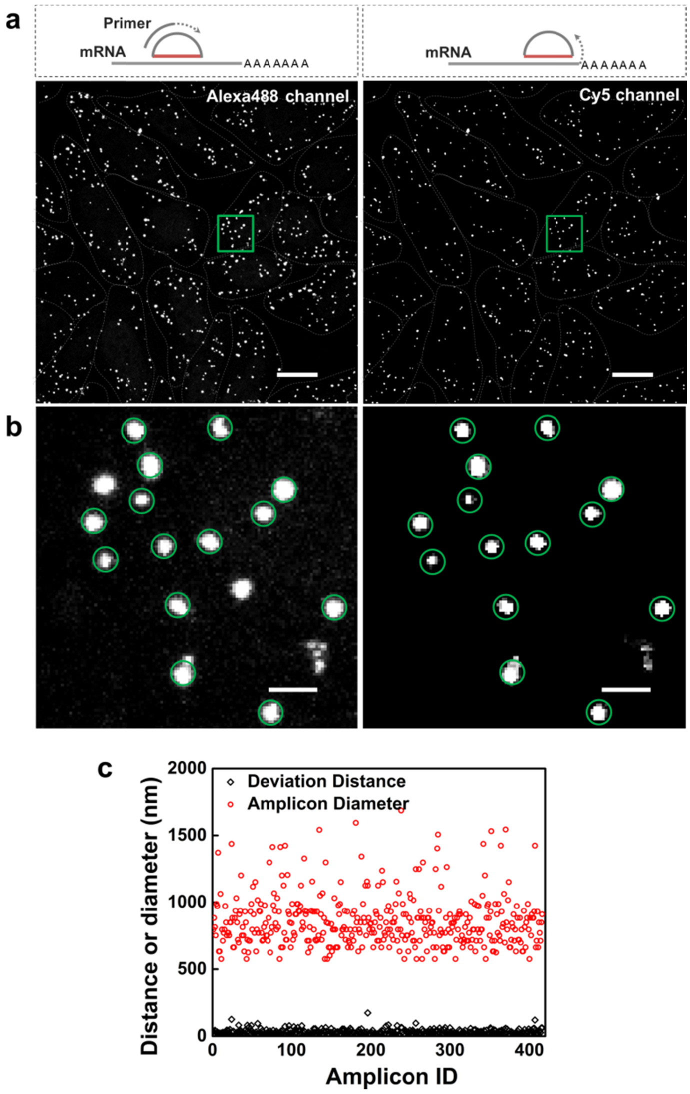

Fig. S5 Co-localization of mRNAs targeted by different sites. (a) Two padlock probes were designed targeting the region in the middle and 3' terminal near poly (A) of mRNA Tk1, respectively. For the padlock probe targeting 3' terminal near poly (A) of mRNA Tk1 (right side), the 3'-5' RNase activity of phi29 DNA polymerase could remove the poly (A) and convert target mRNA into a primer to initiate RCA process.[2] For the padlock probe targeting the middle of mRNA Tk1 (left side), the conversion of target RNA into a primer was hindered. The double-stranded RNA structures present in the 3'-end of the RNA target interfered with the single-stranded 3'-5' RNA exonucleolytic activity.[3] An extra primer was used to perform RCA process. The cell outlines are marked by a dotted line. Scale bar: 20 μm; (b) the amplified image of marked region in (a). Scale bar: 2 μm; (c) the deviation distance of spots in (a) between the two channels, and the diameter of spots in (a) in the alexa488 channel.

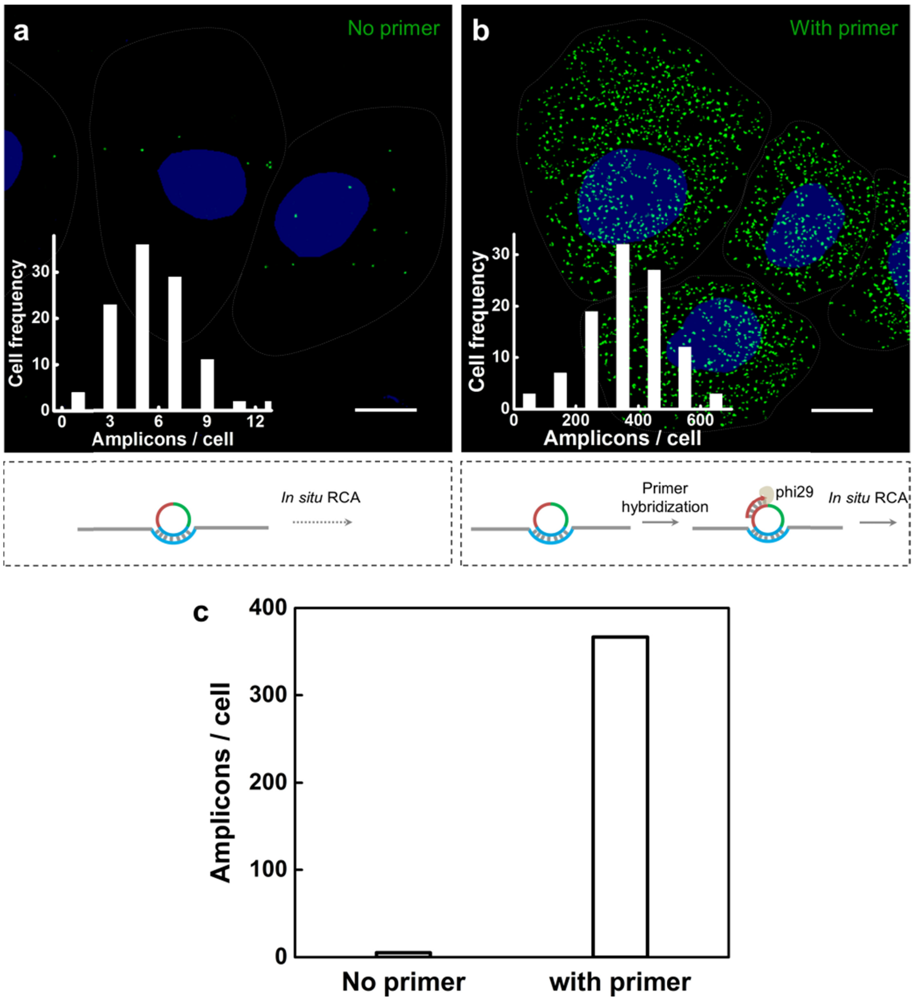

Fig. S6. The effect of the primer on the mRNA detection efficiency. Imaging of mRNA ACTB by target RNA-initiated RCA in MCF-7 cells without primer (a), with primer (b). The green spots represent RCA amplicons hybridized with Cy5-labelled detection probes, the cell nuclei are shown in blue, and the outline of MCF-7 cells is marked in a gray dot line. Scale bars: 10 μm. Inset: frequency histogram of RCA amplicons per cell detected; (c) Quantification of the average number of RCA amplicons per cell detected in (a) and (b).

The detection efficiency of *in situ* amplification and possible improvement. The *in situ* detection efficiency of target RNA-initiated RCA was estimated to be over 20% on the basis of a comparison to RT-qPCR data (Table S3). There are still three main factors that influence the detection efficiency of RCA-based *in situ* mRNA imaging method besides the ligation process. The first effect is the amplification bias of RCA. The secondary structures in target mRNA and padlock probes, or the association with proteins in the mRNA might block the hybridization between the target mRNA and the padlock probes.[4] To reduce the amplification bias, the prediction of structures of target mRNA maybe helpful for avoiding the blocking effect. And the padlock probe should be designed with none or minor secondary structure. Besides, multiple targeting sites on mRNA can be tested for improving the efficiency of hybridization and amplification. The second factor is the relatively low spatial resolution of amplification-based single-molecule imaging method. To provide efficient detection by fluorescence imaging, the RCA amplicons are generally large with diameters of ~1 μm. The formation of hundreds of such RCA amplicons per cell causes the signals to coalesce, limiting the maximum number for digital quantification of target molecules.[5] What's more, the crowding of mRNAs is ubiquitous inside cells such as densely located mRNAs in P-body.[6] These crowed mRNAs render individual mRNA unresolvable, lowering down the detection efficiency. It's the common problem with other single-molecule RNA imaging method like bDNA technology or smFISH. When detecting abundant expressed mRNA ACTB and GAPDH, the detection efficiency of bDNA technology is ~10%, while for the low expressed mRNA the detection efficiency of bDNA is reaching 100%.[7] The third factor may low down the detection efficiency is the imaging process. As the fluorescence images are usually obtained from combing z-sliced images by MIP. The 3D distributed amplicons were coalesced in the flattening process,

making the z-axis segregate amplicons unresolvable. Besides to distinct a single amplicon from the background signal by setting the intensity threshold, some amplicons with relatively dim fluorescence may be lost. In conclusion, there's much room for improving the detection efficiency of RCA based mRNA imaging method. Nevertheless, at present, target RNA-initiated RCA using Splint R as ligase is with relatively high detection efficiency (over 20%), which is suitable for quantifying or relative expression profiling of mRNAs in single cells.

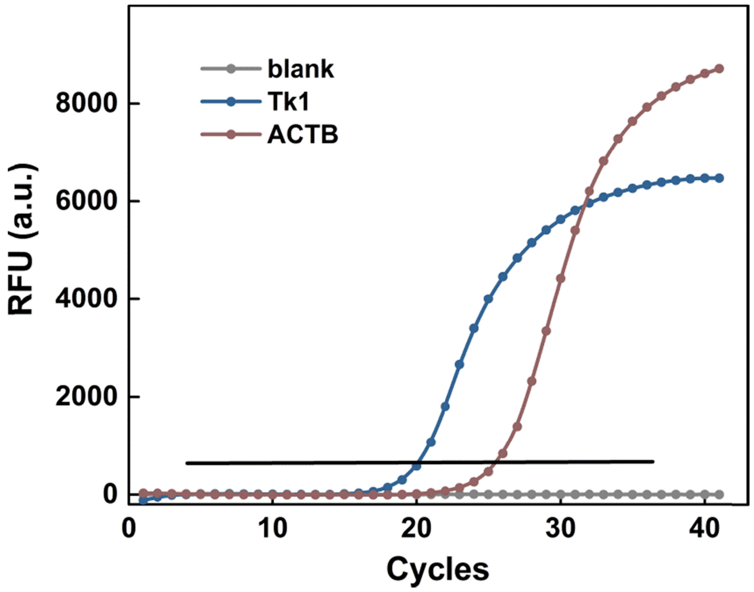

Fig. S7. Expression analysis of target mRNAs in the MCF-7 cells by RT-qPCR. Real-time fluorescence curves in RT-qPCR analysis. The black horizontal line represents the threshold line. Each sample was detected in three repetitive assays.

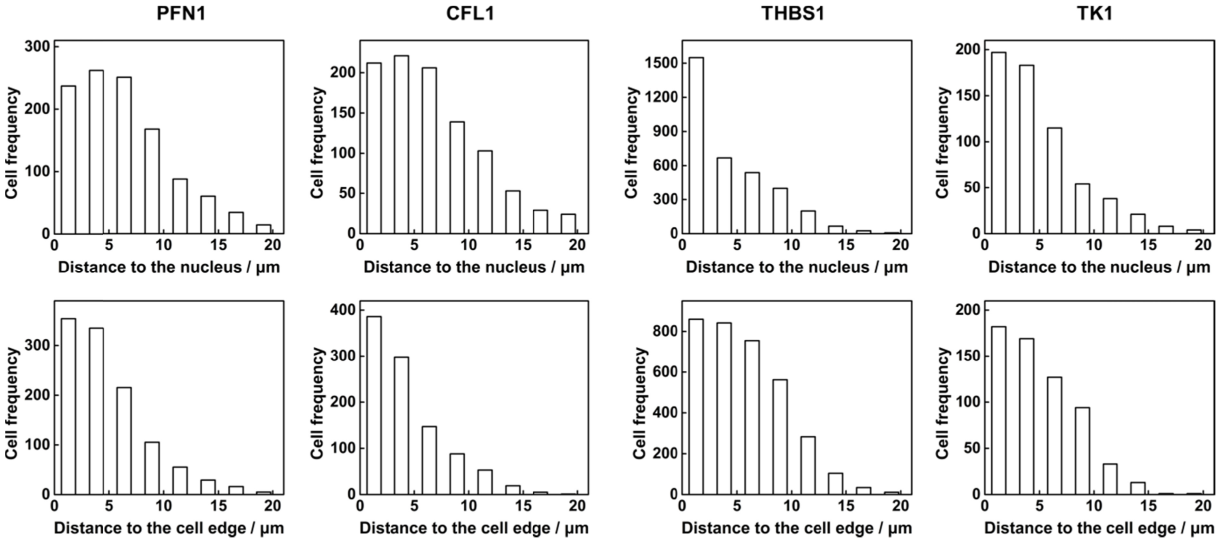

Fig. S8. Histograms of the distances for the mRNAs PFN1, CFL1, THBS1 and TK1 to the nucleus and the cell edge of each MCF-7 cells (over 100 cells checked).

Table S3. The average numbers of ACTB and TK1 in the MCF-7 cells measured by target RNA-initiated RCA and RT-qPCR

Target mRNA	Ligase for mRNA detection	Average number measured (copies / cell)	C_t	Average number measured by qPCR (copies / cell)	Detection efficiency (%)
ACTB	T4 DNA ligase	210.51	20.18	1929.38	10.91
	T4 RNA ligase 2	187.18			9.70
	Splint R	380.71			19.73
TK1	T4 DNA ligase	14.87	25.56	102.79	14.47
	T4 RNA ligase 2	14.03			13.65
	Splint R	28.50			27.73

References

1 K. J. Livak and T. D. Schmittgen, *Methods,* 2001, **25**, 402-408.

2 A. Lagunavicius, E. Merkiene, Z. Kiveryte, A. Savaneviciute, V. Zimbaite-Ruskuliene, T. Radzvilavicius and A. Janulaitis, *RNA,* 2009, **15**, 765-771.

3 E. Merkiene, E. Gaidamaviciute, L. Riauba, A. Janulaitis and A. Lagunavicius, *RNA,* 2010, **16**, 1508-1515.

4 R. Ke, M. Mignardi, A. Pacureanu, J. Svedlund, J. Botling, C. Wählby and M. Nilsson, *Nat. Methods,* 2013, **10**, 857-860.

5 C. M. Clausson, A. Allalou, I. Weibrecht, S. Mahmoudi, M. Farnebo, U. Landegren, C. Wählby and O. Söderberg, *Nat. Methods,* 2011, **8**, 892-893.

6 T. M. Franks and J. Lykke-Andersen, *Mol. Cell,* 2008, **32**, 605-615.

7 N. Battich, T. Stoeger and L. Pelkmans, *Nat. Methods,* 2013, **10**, 1127-1133.
